# Supplementary material for: Mapping the evolution of fertility support policies in China: A content and instrumental analysis
Source: PLoS One. 2025 Oct 9;20(10):e0332137. doi: 10.1371/journal.pone.0332137 (PMC12510515; doi:10.1371/journal.pone.0332137)
Supplement: S1 Appendix — (ZIP) [file pone.0332137.s001.zip › S1 Appendix. 226 original policy documents/114-人力资源社会保障部、财政部关于适当降低生育保险费率的通知(FBM-CLI-4-252303).doc]

人力资源社会保障部、财政部关于适当降低生育保险费率的通知

**发布部门：** [人力资源和社会保障部](https://www.pkulaw.com/law/chl?Aggs.IssueDepartment=60236) [财政部](https://www.pkulaw.com/law/chl?Aggs.IssueDepartment=60210) [机构沿革](javascript:void(0))

**发文字号：**人社部发〔2015〕70号

**发布日期：**2015.07.27

**实施日期：**2015.10.01

**时效性：** [现行有效](https://www.pkulaw.com/law/chl?Aggs.TimelinessDic=01)

**效力级别：** [部门规范性文件](https://www.pkulaw.com/law/chl?Aggs.EffectivenessDic=XE0302)

**法规类别：** [保险综合规定](https://www.pkulaw.com/law/chl?Aggs.Category=04402)

人力资源社会保障部、财政部关于适当降低生育保险费率的通知
（人社部发〔2015〕70号）

各省、自治区、直辖市人力资源社会保障厅（局）、财政厅（局），新疆生产建设兵团人力资源社会保障局、财务局：
　　按照党的十八届三中全会提出的“适时适当降低社会保险费率”的精神，根据生育保险基金实际情况，经国务院同意，自2015年10月1日起，在生育保险基金结余超过合理结存的地区降低生育保险费率。现就有关问题通知如下：
　　**一、**统一思想，提高认识，确保政策落到实处
　　各地生育保险制度建立以来，在促进女性平等就业，均衡用人单位负担，维护女职工权益等方面发挥了重要作用。但也存在着地区间发展不平衡，基金结余偏多，待遇支付不规范等方面的问题。对基金结余多的地区降低生育保险费率，是完善生育保险政策，提高基金使用效率的一个重大举措，也是进一步减轻用人单位负担，促进就业稳定，实施积极财政政策的具体体现。各地要统一思想，充分认识降低生育保险费率的重要意义，确保政策按时落实到位，取得实效。

　　**二、**认真测算，降低费率，控制基金结余
　　生育保险基金合理结存量为相当于6至9个月待遇支付额。各地要根据上一年基金收支和结余情况，以及国家规定的待遇项目和标准进行测算，在确保生育保险待遇落实到位的前提下，通过调整费率，将统筹地区生育保险基金累计结余控制在合理水平。生育保险基金累计结余超过9个月的统筹地区，应将生育保险基金费率调整到用人单位职工工资总额的0.5%以内，具体费率应按照“以支定收、收支平衡”的原则，根据近年来生育保险基金的收支和结余情况确定。
　　各地要加强对生育保险基金的监测和管理。降低生育保险费率的统筹地区要按程序调整生育保险基金预算，按月进行基金监测。基金累计结余低于3个月支付额度的，要制定预警方案，并向统筹地区政府和省级人力资源社会保障、财政部门报告。要通过提高统筹层次，加强基金和医疗服务管理，规范生育保险待遇，力求基金平衡。在生育保险基金累计结余不足支付时，统筹地区要采取加强支出管理、临时补贴、调整费率等方式确保基金收支平衡，确保参保职工按规定享受生育保险待遇。

　　**三、**加强组织领导，全面推进实施
　　各省（区、市）人力资源社会保障、财政部门要加强配合，共同研究落实国务院降低生育保险费率措施。实行省级统筹且基金结余超过9个月的省（区、市），应于9月底前提出降低生育保险费率的办法，报省级人民政府批准后实施。未实行省级统筹的省（区、市），应于8月底前制订本省（区、市）降低生育保险费率的办法，指导各统筹地区制订实施方案，符合降费率规定的统筹地区应于9月底以前发布降低费率的实施方案，以确保10月1日前完成降低生育保险费率的工作。各省（区、市）应于9月底将上述情况报告人力资源社会保障部、财政部。
　　要加强降低生育保险费率的宣传工作，向工作人员、参保单位和广大职工讲清降低生育保险费率的重大意义，在减轻用人单位负担的同时，调动用人单位参保积极性，切实维护女职工合法权益。要加强与有关部门协调配合，做好人口出生形势的分析和预判。各地在政策调整过程中出现的新情况、新问题，要及时与人力资源社会保障部、财政部进行沟通，采取有效措施，确保工作落实到位。

人力资源社会保障部
财政部
2015年7月27日

© 北大法宝：（[www.pkulaw.com](https://www.pkulaw.com/)) 专业提供法律信息、法学知识和法律软件领域各类解决方案。北大法宝为您提供丰富的参考资料，正式引用法规条文时请与标准文本核对。
欢迎查看所有[产品和服务](https://www.pkulaw.com/helps/2.html)。法宝快讯：[如何快速找到您需要的检索结果？](https://www.pkulaw.com/helps/70.html)[法宝](https://www.pkulaw.com/helps/69.html)V6有何新特色？

扫描二维码阅读原文

**原文链接：**<https://www.pkulaw.com/chl/7fc6684be48ccee9bdfb.html>
